# Cholestasis induces reversible accumulation of periplakin in mouse liver

**DOI:** 10.1186/1471-230X-13-116

**Published:** 2013-07-13

**Authors:** Shinji Ito, Junko Satoh, Tsutomu Matsubara, Yatrik M Shah, Sung-hoon Ahn, Cherie R Anderson, Weiwei Shan, Jeffrey M Peters, Frank J Gonzalez

**Affiliations:** 1 Biofrontier Platform, Graduate School of Medicine, Kyoto University, Yoshida Konoe-cho, Sakyo-ku, Kyoto 606-8501, Japan; 2 Department of Anatomy and Regenerative Biology, Graduate School of Medicine, Osaka City University, 1-4-3 Asahi-machi, Abeno-ku, Osaka 545-8585, Japan; 3 Department of Molecular and Integrative Physiology, University of Michigan, 7712B Med Sci II, 1137 E. Catherine St., Ann Arbor, MI 48109-5822, USA; 4 Drug Discovery Platform Technology Team, Korea Research Institute of Chemical Technology, Sinseongno 19, Yoosung, Daejeon 305-343, South Korea; 5 Department of Veterinary and Biomedical Sciences and the Center for Molecular Toxicology and Carcinogenesis, Pennsylvania State University, 312 Life Sciences Building, University Park 16802, Pennsylvania, USA; 6 Department of Pathology, University of Texas MD Anderson Cancer Center, 1515 Holcombe Blvd., Houston, Texas 77030, USA; 7 Laboratory of Metabolism, Center for Cancer Research, National Cancer Institute, National Institutes of Health, Building 37, Room 3106, 37 Convent Drive, Bethesda, Maryland 20892, USA

**Keywords:** Periplakin, Cholestasis, Bile acids, Farnesoid X receptor, Urinary stasis

## Abstract

**Background:**

Periplakin (PPL) is a rod-shaped cytolinker protein thought to connect cellular adhesion junctional complexes to cytoskeletal filaments. PPL serves as a structural component of the cornified envelope in the skin and interacts with various types of proteins in cultured cells; its level decreases dramatically during tumorigenic progression in human epithelial tissues. Despite these intriguing observations, the physiological roles of PPL, especially in non-cutaneous tissues, are still largely unknown. Because we observed a marked fluctuation of PPL expression in mouse liver in association with the bile acid receptor farnesoid X receptor (FXR) and cholestasis, we sought to characterize the role of PPL in the liver and determine its contributions to the etiology and pathogenesis of cholestasis.

**Methods:**

Time- and context-dependent expression of PPL in various mouse models of hepatic and renal disorders were examined by immunohistochemistry, western blotting, and quantitative real-time polymerase chain reactions.

**Results:**

The hepatic expression of PPL was significantly decreased in *Fxr*^−/−^ mice. In contrast, the expression was dramatically increased during cholestasis, with massive PPL accumulation observed at the boundaries of hepatocytes in wild-type mice. Interestingly, the hepatic accumulation of PPL resulting from cholestasis was reversible. In addition, similar accumulation of PPL at cellular boundaries was found in epithelial cells around renal tubules upon ureteral obstruction.

**Conclusions:**

PPL may be involved in the temporal accommodation to fluid stasis in different tissues. Further examination of the roles for PPL may lead to the discovery of a novel mechanism for cellular protection by cytolinkers that is applicable to many tissues and in many contexts.

## Background

Hepatic bile production is required for the excretion of cholesterol and other waste products into the small intestine. When bile flow is obstructed, biliary constituents re-enter the liver and the systemic circulation, causing a variety of local and systemic disorders, including jaundice, pruritus, fatigue, osteoporosis, malabsorption, abnormal lipid metabolism, and immunosuppression [1]. In addition, protracted cholestasis can elicit hepatic fibrosis, which could eventually result in hepatic cirrhosis, hepatocarcinogenesis, and loss of liver function [1]. Bile acids, the major constituents of bile, play critical roles in many of these derangements through their detergent-like properties or the activation of their receptors farnesoid X receptor (FXR, NR1H4) and G-protein-coupled bile acid receptor 1 (GPBAR1, also known as Takeda G-protein-coupled receptor 5) [2,3]. However, it is difficult to fully explain the pathogenesis of cholestasis and concomitant disorders by the direct actions of bile acids or the actions mediated through the already known molecules controlled by FXR and GPBAR1 alone; therefore, additional regulatory molecules likely exist.

To discover the additional regulatory molecules engaged in the pathogenesis of cholestasis and concomitant disorders, we sought previously undescribed molecules associated with FXR and cholestasis, because evidence exists that FXR critically influences the pathophysiology of cholestasis in mouse models [4-6] and human familial intrahepatic cholestasis type 1 (also known as Byler's disease) [7]. Examination of hepatic gene expression profiles of *Fxr* null (*Fxr*^−/−^) and wild-type mice [8], and comparison with the expression of selected genes in a cholestatic mouse model (dietary cholic acid [CA]-administration), revealed that periplakin (PPL) is a unique protein central to FXR and cholestasis. The basal expression level of *Ppl* mRNA was reduced in the liver of *Fxr*^−/−^ mice, whereas it was markedly increased during cholestasis in wild-type mice. To our knowledge, this is the first study that showed a relationship among FXR, cholestasis, and PPL.

PPL is a member of the family of plakin/cytolinker proteins, which connect cellular adhesion junctional complexes to cytoskeletal filaments. Among the plakin family proteins, PPL stands out for its size (the smallest in the plakin family) and atypical domain structures (lacks the actin-binding domain and proline-rich domain that are conserved in most of plakin family proteins) [9-11]. Historically, PPL was first discovered in the 1980s as one of the constituents of the cornified envelope (CE), the outermost layer of the epidermis that provides a physical barrier for the skin [12]. In the 1990s, PPL cDNA was isolated from keratinocytes [13,14]. Since its initial identification, the role of PPL has been studied primarily in the skin, despite its expression in many other tissues [13-15]. During keratinocyte differentiation into corneocytes, PPL forms a heterodimer with another plakin, envoplakin (EVPL), and is covalently cross-linked to the heterogeneous network of desmosomes (macula adherens), keratin filaments, loricrin, involucrin (IVL), small proline-rich proteins, and membrane lipids via the enzymatic activities of transglutaminases (TGases). These molecular changes result in flattening and stacking of cornified cells to form the CE layer of the epidermis [16].

Interestingly, the heterodimeric partner for PPL, EVPL, is predominantly expressed in the skin, kidney, lung, and prostate [15,17]. Thus, PPL has EVPL-independent functions in other tissues, including the liver. Indeed, many *in vitro* studies have revealed that PPL may modulate the intracellular cytoskeletal network through interaction with a number of molecules other than EVPL, including vimentin, keratin 8 (K8), beaded filament structural component 2 (also known as cytoskeletal protein 49 kDa), filensin, plectin, and collagen type XVII (also known as bullous pemphigoid antigen 2) [14,15,18,19]. PPL has also been shown to inhibit intracellular signal transduction through interaction with family A (rhodopsin-like) G-protein-coupled receptors [20-22]. Interactions between PPL and Akt (also known as protein kinase B) and the intracellular tail of immunoglobulin G receptor (FcγRI) have also been reported [23,24]. Thus, PPL may serve not only as a structural component in cells but also as a functional regulatory molecule in various tissues.

Recently, studies in cancer proteomics have revealed that PPL expression is markedly reduced during the progression of human esophageal squamous cell carcinoma [25,26]. This decreased expression of PPL, which closely paralleled the progression of malignancy, was associated with changes in the intracellular localization of PPL from the cellular periphery to the inner cytosol [25]. A similar reduction in PPL expression was also reported in urothelial cells during the progression of bladder cancer [27]. Despite these intriguing observations in previous studies, *Ppl* null (*Ppl*^−/−^) mice did not exhibit an overt phenotype under normal breeding conditions [28]. On the other hand, *Ivl*/*Evpl*/*Ppl* triple-null mice exhibited defective epidermal barrier formation due to an abnormal assembly of CE-layer components, suggesting the functional redundancy in these 3 proteins in the skin [29].

Taken together, these studies suggest that PPL acts as a structural component in the skin, interacts with various types of proteins *in vitro*, and may be involved in tumorigenic progression in human epithelial tissues; however, the physiological roles for PPL, especially in non-cutaneous tissues, including the liver, remain uncertain. In this study, we examined the expression of PPL in the liver of *Fxr*^−/−^ mice and in various cholestatic mouse models in detail. The clarified pattern of expression of PPL in these mice suggested that PPL plays an important role in the temporal accommodation to cholestasis. The possible roles for PPL in urinary stasis and tumorigenesis are also discussed.

## Methods

### Animals

Two- to 3-month-old male C57BL/6JJcl mice (CLEA, Tokyo, Japan) fed an NMF diet (Oriental Yeast, Tokyo, Japan) were used for all experiments, unless otherwise specified. *Fxr*^+/+^ and *Fxr*^−/−^ mice [8] were fed a Rodent NIH-31 Auto 18–4 diet (Zeigler Brothers, Gardner, PA, USA) and sacrificed at 2–3 months of age. Five-week-old male leptin-deficient genetically obese *ob*/*ob* or the control +/+ mice (C57BL/6JHamSlc-*ob*/*ob* or +/+; SLC, Hamamatsu, Japan) were used after acclimation to the NMF diet for 2 weeks. All mice were fed *ad libitum* and housed in a light- and temperature-controlled room under specific-pathogen-free conditions. Mice were euthanized by cervical dislocation at the end of each treatment. All animal studies were approved by the relevant animal care and use committees of Kyoto University, National Cancer Institute (NCI), and Pennsylvania State University (PSU), and were carried out in accordance with the Guide for the Care and Use of Laboratory Animals published by the National Institutes of Health (NIH).

### *Ppl* null mice

*Ivl*/*Evpl*/*Ppl* triple-heterozygous null (*Ivl*^+/−^;*Evpl*^+/−^;*Ppl*^+/−^) mice were produced by the mating of *Ivl*/*Evpl*/*Ppl* triple-homozygous null (*Ivl*^−/−^/*Evpl*^−/−^/*Ppl*^−/−^) mice [29] (kindly provided by Drs. Fiona Watt [Cancer Research UK], Jouni Uitto [Thomas Jefferson University], and John F. Klement [Thomas Jefferson University]) with C57BL/6JJcl mice. All of the single-homozygous null (*Ivl*^−/−^, *Evpl*^−/−^, and *Ppl*^−/−^) mice used for the creation of the triple-homozygous null mice had been backcrossed onto a C57BL/6 background in the different facilities at which they were generated [29]. Triple-heterozygous null offspring were further backcrossed onto the C57BL/6JJcl background for 2 more generations to obtain *Ppl* single-heterozygous null (*Ppl*^+/−^ [*Ivl*^+/+^/*Evpl*^+/+^/*Ppl*^+/−^]) mice. *Ppl*^+/+^ and *Ppl*^−/−^ mice born from mating of *Ppl*^+/−^ mice were used in this study. These mice have been backcrossed a total of 3 times onto the C57BL/6JJcl background.

### Serum chemistry

Blood was collected by retro-orbital bleeding. The sera were separated by centrifugation at 3000 rpm for 10 min. Serum total bile acid (T-BA) levels were determined using the Total Bile Acid Assay kit (Diazyme Laboratories, Poway, CA, USA). Other parameters were determined by Oriental Bioservice, Inc. (Kyoto, Japan).

### Chronic dietary treatments

Mice were fed the control NMF diet or the same diet supplemented with 0.1%, 0.25%, or 1% w/w CA (Sigma, St. Louis, MO, USA) for 7 days. Sodium lauryl sulfate (SDS; Nacalai Tesque, Kyoto, Japan) was administered in drinking water at 0.1%, 0.25%, or 1% (w/v) for 7 days. *Fxr*^+/+^ or *Fxr*^−/−^ mice fed the Rodent NIH-31 Auto 18–4 diet were subjected to a dietary switch at the age of 2 months; control NMF or 1% CA/NMF diet was provided for an additional 7 days.

### Acute administration of bile acids and synthetic FXR ligands

CA or GW4064 (Tocris, Ellisville, MO, USA) dissolved in 1% Tween 80/1% methylcellulose or the corresponding vehicle solutions was dosed twice orally at 0, 10, 100, or 1000 mg/kg (12-h interval; first administration at 2000 and second administration at 0800). Mice were euthanized 2 h after the second administration.

### Serial administration of the high-fat diet (HFD) and the CA-containing diet

Mice fed the NMF diet were administered a HFD (#F3282; Bioserv, Frenchtown, NJ, USA) for 8 weeks. The mice were then subjected to a dietary switch from the HFD to the 1% CA-containing NMF or the control NMF diet for an additional 7 days.

### ConA treatment

Mice were given 20 mg/kg concanavalin A type IV (ConA; Sigma) dissolved in saline or the volume-matched vehicle solution by tail vein injection. Mice were euthanized 8, 24, or 48 h after the injection.

### CCl_4_ treatment

Two- to 3-month-old male C57BL/6 mice were injected intraperitoneally with carbon tetrachloride (CCl_4_) at 1 mL/kg dissolved in corn oil [30]. For the short-term treatment, mice were euthanized 24 or 48 h after the injection. For long-term treatment, the mice were administered the same dose twice a week for 4 weeks. Control mice were administered volume-matched corn oil.

### ANIT treatment

α-Naphthylisothiocyanate (ANIT; Aldrich, Milwaukee, WI, USA) at 50 mg/kg dissolved in olive oil or volume-matched vehicle solution was orally administered after overnight fasting. Mice were euthanized 48 h after the administration.

### Bile duct ligation (BDL) and unilateral ureteral obstruction (UUO)

Mice were anesthetized by intraperitoneal injection of Avertin. After midline laparotomy, the common bile duct (for BDL) or right ureter (for UUO) was double-ligated with 8–0 nylon or 4–0 silk suture, respectively. Sham operations were carried out by the same procedures in which the ligation was substituted by gentle touching of the bile duct or ureter. Mice were sacrificed 2, 7, or 21 days after the surgery.

### Immunohistochemistry

Sections (6 μm) from frozen tissues mounted in Tissue-Tek O.C.T. compound (Sakura Finetek, Tokyo, Japan) were attached to MAS-coated slide glasses (Matsunami Glass, Osaka, Japan), fixed in methanol for 10 min at −20°C, and permeabilized with 0.1% Triton X-100 in PBS for 5 min. After blocking with 1% bovine serum albumin (BSA)/PBS for 1 h, sections were incubated with primary antibodies diluted in 1% BSA/PBS overnight at 4°C. The following antibodies were used: anti-PPL (A301-005A; Bethyl Laboratories, Montgomery, TX, USA), anti-keratin 19 (K19; TROMAIII; Developmental Studies Hybridoma Bank, Iowa, IA, USA), anti-zonula occludens-1 (ZO-1; sc-33725; Santa Cruz Biotechnology, Santa Cruz, CA, USA), anti-multidrug-resistance protein (MDR; sc-8313; Santa Cruz Biotechnology), anti-γ-catenin (γ-CTN; sc-30996; Santa Cruz Biotechnology), anti-aquaporin 2 (AQP2; sc-9882; Santa Cruz Biotechnology), and anti-K8 (ab14053; Abcam, Cambridge, MA, USA). Slides were washed with PBS 3 times and incubated with Alexa488- or Alexa594-conjugated secondary antibodies (Molecular Probes, Eugene, OR, USA) at room temperature for 1 h or 4°C overnight. Slides were then washed with PBS, mounted in Fluoromount (Diagnostic Biosystems, Pleasanton, CA, USA), and examined using fluorescence microscopy (DMRBE; Leica Microsystems, Tokyo, Japan). Staining patterns for PPL were confirmed using peptide blocking (BP301-005; Bethyl Laboratories), an alternative antibody (customized polyclonal antibody raised against mouse PPL; MBL, Nagoya, Japan), and *Ppl*^−/−^ mice. Staining patterns for K19 were also confirmed by use of an alternative antibody (ab52625; Abcam).

### Western blots

Frozen tissues were homogenized in protein extraction buffer (50 mM Tris–HCl, 5 mM EDTA, 2% SDS, 10% glycerol, 10 mM NaF, 1 mM Na_3_VO_4_) supplemented with Complete Protease Inhibitor Cocktail (Roche, Indianapolis, IN, USA) using a Physcotron (Microtec Nition, Funabashi, Japan). Aliquots (15 μg) of samples were separated on 5–20% polyacrylamide gradient gels, transferred to a polyvinylidene difluoride membranes (Immobilon-P; Millipore, Billerica, MA, USA), and hybridized with primary antibodies diluted in Tris-buffered saline containing 6% skim milk and 0.05% Tween-20 (6% skim milk/TBST) overnight at 4°C. Membranes were washed with 0.05% Tween-20-containing Tris-buffered saline (TBST) 3 times and hybridized with horseradish peroxidase-conjugated secondary antibodies diluted in 6% skim milk/TBST at room temperature for 1 h or 4°C overnight. After washing with TBST 3 times, signals were detected by an ECL Plus western blotting detection system (GE Healthcare, Chalfont St. Giles, UK). The immunoblots for glyceraldehyde-3-phosphate dehydrogenase (GAPDH) were used as the loading controls. The following antibodies were used: PPL (A301-005A; Bethyl Laboratories), K19 (TROMAIII; Developmental Studies Hybridoma Bank), ZO-1 (sc-33725; Santa Cruz Biotechnology), γ-CTN (2309; Cell Signaling Technology, Danvers, MA, USA), DSG1 (sc-2011; Santa Cruz Biotechnology), K8 (ab14053; Abcam), K18 (ab32118; Abcam), plectin (ab32528; Abcam), and GAPDH (2118; Cell Signaling Technologiy). For PPL, K19, and ZO-1, the results were confirmed by the use of alternative antibodies.

### NCI oligo-cDNA microarray

Equal amounts of total RNA samples extracted from the liver of 2- to 3-month-old male *Fxr*^+/+^ and *Fxr*^−/−^ mice, using TRIZOL Reagent (Invitrogen, Carlsbad, CA, USA), were pooled for each genotype. Amino-modified cDNAs were synthesized from 20 μg of pooled RNAs by using a SuperScript indirect cDNA labeling kit (Invitrogen) and labeled with Cy3 or Cy5 fluorescent dyes (Amersham Pharmacia, Piscataway, NJ, USA). Dye-coupled cDNAs were hybridized to the NCI in-house printed oligo-DNA spotted glass slides (Mouse Exotic Evidence Based Oligonucleotide Set, Gene Expression Omnibus accession number GPL4200). The slides were analyzed using a GenePix 4000A dual-laser scanning system and GenePix Pro 3.0 (Axon Instruments, Foster City, CA, USA). Datasets were further analyzed by the microarray Database system provided by the Center for Information Technology at the NIH.

### Quantitative RT-PCR (qPCR)

Total RNA (1 μg) extracted using TRIZOL Reagent was pretreated with DNaseI (Invitrogen) and reverse-transcribed by Superscript III reverse transcriptase (Invitrogen) and primer p(dT)_15_ (Roche). Real-time PCR was performed using an ABS 7300 sequence detection system or StepOnePlus real-time PCR system (Applied Biosystems, Foster City, CA, USA) with RT^2^ Real-Time SYBR Green/Rox PCR master mix (SuperArray Biosciences, Frederick, MD, USA) according to the manufacturer’s instruction. The real-time PCR conditions were 95°C for 10 min, followed by 40 cycles of 95°C for 15 s and 60°C for 1 min. Absence of primer-dimers were confirmed after reactions by melting curve analysis and agarose gel electrophoresis. Relative expression levels were determined by the ΔΔCt method using *Gapdh* or *36b4* mRNA (for *ob*/*ob* mice) levels as internal controls. These control primers yielded constant values over the treatments in the same tissues or cells.

### Mouse primary hepatocytes

Mice were anesthetized with 0.5% sodium pentobarbital, and livers were digested with a 2-step perfusion method using liver perfusion and digestion media (Invitrogen) delivered through the portal vein. Recovered cells were washed 3 times with plating medium (Dulbecco’s modified Eagle medium supplemented with 5% fetal bovine serum and penicillin-streptomycin) and resuspended in the same medium. The live cells were seeded onto collagen IV-coated 6-well plates (BD, Franklin Lakes, NJ, USA) at 3.5 × 10^5^ cells per well. Three hours after the seeding, the medium was replaced with serum-free medium (Medium199 supplemented with 0.1 μM dexamethasone, 0.1 μM insulin, and 0.1 μM T3) containing bile acids or synthetic FXR ligand at various concentrations. Bile acids (CA, chenodeoxycholic acid [CDCA], deoxycholic acid [DCA], and lithocholic acid [LCA]; all from Sigma) and GW4064 were dissolved in dimethyl sulfoxide (DMSO) and used at 0.1% final DMSO concentration in culture. The cells were harvested 20 h after ligand treatment.

### Statistical analysis

Datasets were evaluated by 2-tailed unpaired *t* test, Mann–Whitney *U* test, Steel’s test, or one-way analysis of variance (ANOVA) with *post hoc* tests (Dunnett’s or Bonferroni’s test) for statistical significance using Prism 5 (GraphPad Software, San Diego, CA, USA) and MEPHAS (http://www.gen-info.osaka-u.ac.jp). *P* values less than 0.05 were considered significant.

## Results

### *Ppl* mRNA expression was reduced in the liver of *Fxr*^−/−^ mice and markedly increased during cholestasis in wild-type mice

**Figure 1:**
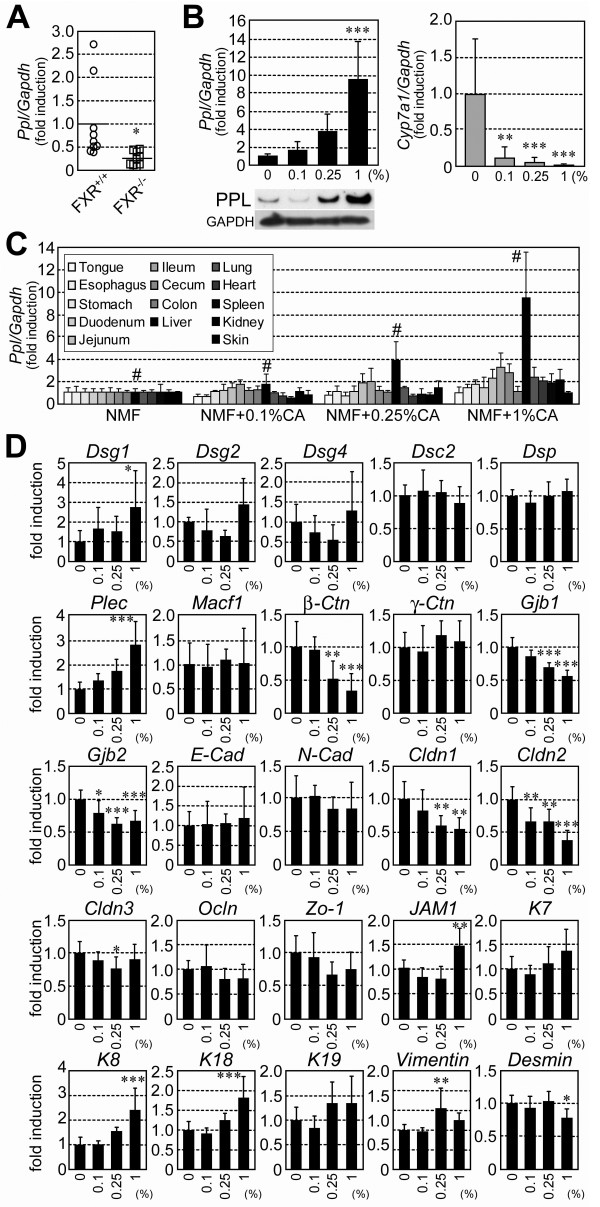
**PPL expression in *Fxr***^**−/− **^**and CA-fed cholestasis model mice.** (**A**) Hepatic *Ppl* mRNA expression levels in *Fxr*^+/+^ and *Fxr*^−/−^mice. Seven or 8 mice per group were examined. Circles and rectangles correspond to individual animals. Horizontal bars represent averages. (**B**) Hepatic *Ppl* and *Cyp7a1* mRNA expression levels in wild-type mice fed cholic acid (**CA**) for 7 days at the indicated doses. Five to 9 mice per group were examined. The immunoblot for PPL is also shown. Equal amounts of protein from 5 mice were combined in each group for western blotting. The immunoblot for GAPDH was used as the loading control. (**C**) *Ppl* mRNA expression in various tissues following CA feeding for 7 days. Bars for each tissue are grayscaled as indicated (darkened from left to right, tongue to skin). Bars for the liver are flagged with a number sign (#). Four samples per group were examined. (**D**) Hepatic mRNA expression of plakins, cell junction proteins, and intermediate filaments in wild-type mice fed CA for 7 days. Five to 9 mice per group were examined. mRNA expression levels are represented as values normalized to *Gapdh*. The average values in *Fxr*^+/+^ and control diet-fed mice are set as 1.0. Values represent mean ± SD. *Dsg1*/*2*/*4*, *desmoglein*-*1*/-*2*/-*4*; *Dsc2*, *desmocollin*-*2*; *Dsp*, *desmoplakin*; *Plec*, *plectin*; *Macf1*, *microtubule actin crosslinking factor*-*1*; *β*/*γ*-*Ctn*, *β*/*γ*-*catenin*; *Gjp1*/*2*, *gap junction protein*-*1*/-*2*; *E*/*N*-*Cad*, *E*/*N*-*cadherin*; *Cldn1*/*2*/*3*, *claudin*-*1*/-*2*/-*3*; *Ocln*, *occludin*; *Zo*-*1*, *zonula occludens*-*1*; *JAM1*, *junctional adhesion molecule*-*1*; *K7*/*8*/*18*/*19*, *keratin 7*/*8*/*18*/*19*. *, **, ***: *P* < 0.05, 0.01, and 0.001, respectively, by Mann–Whitney *U* test (A) or one-way ANOVA with Dunnett’s test (B and D [vs. 0%]).

Hepatic gene expression profiles of *Fxr*^+/+^ and *Fxr*^−/−^ mice were compared by cDNA microarray to identify genes constitutively regulated by FXR in the liver. Among the genes that appeared to be regulated by FXR, we focused on the gene encoding PPL, a plakin/cytolinker protein. Hepatic *Ppl* mRNA expression was considerably reduced in *Fxr*^−/−^ mice (8-fold). The reduction was confirmed by qPCR analysis. Indeed, expression level of *Ppl* mRNA in the liver of *Fxr*^−/−^ mice was typically lower than that in the liver of *Fxr*^+/+^ mice (Figure 1A). To our knowledge, this is the first molecule in this family whose expression is associated with FXR. Expression of PPL during cholestasis induced by the administration of CA through the diet (0.1%–1% for 7 days) was also evaluated in the liver of wild-type mice. PPL expression was markedly increased in a dose-dependent manner at both the mRNA and protein levels in response to dietary CA (Figure 1B, *left*). Expression of the cholesterol 7α-hydroxylase gene (cytochrome P450 7a1 [*Cyp7a1*]) was suppressed even at the lowest concentration of dietary CA, confirming activation of FXR (Figure 1B, *right*). Interestingly, the CA-dependent induction of *Ppl* mRNA expression was highly tissue selective. Among the tissues examined, including those directly or indirectly exposed to dietary CA, the induction was particularly prominent in the liver, suggesting an important role for PPL in the liver (Figure 1C). The regulation of *Ppl* mRNA expression in the liver, following CA feeding, was unique for its robustness, as compared to other plakins and representative components of cytoskeleton and junctional complexes (Figure 1D).

### PPL was localized to the cholangiocytes and the boundaries of hepatocytes in normal liver

**Figure 2:**
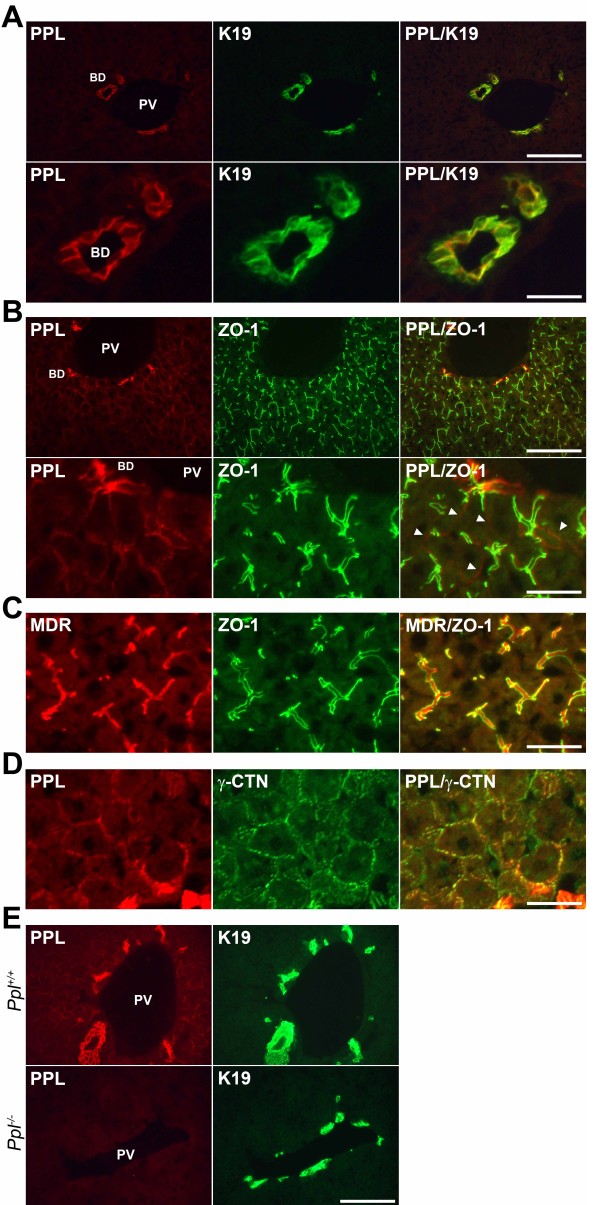
**Immunohistochemical localization of PPL in normal liver.** (**A**) Representative images for the localization of PPL and K19 in normal liver. PPL and K19 were double-stained by red and green fluorescence, respectively. Merged images are shown on the right. Scale bars: 100 μm (*upper*) and 25 μm (*lower*). (**B**) PPL and ZO-1 were double-stained by red and green fluorescence, respectively. Merged images are shown on the right. Arrowheads indicate PPL expression at ZO-1-negative sites. Scale bars: 100 μm (*upper*) and 25 μm (*lower*). (**C**) MDR and ZO-1 were double-stained by red and green fluorescence, respectively. Merged images are shown on the right. Scale bar: 25 μm. (**D**) PPL and γ-catenin (γ-CTN) were double-stained by red and green fluorescence, respectively. Merged images are shown on the right. Scale bar: 25 μm. (**E**) PPL and K19 were double-stained by red and green fluorescence, respectively. Signals for PPL were absent in cholangiocytes and hepatocytes of *Ppl*^−/−^ mice. Scale bar: 100 μm. PV, portal vein; BD, bile duct.

Next, we determined the localization of PPL in the liver of wild-type mice by immunohistochemical analysis. The highest expression of PPL was detected in bile duct epithelial cells co-expressing K19, a cholangiocyte marker (Figure 2A). PPL was also detected at the boundaries of hepatocytes and overlapped with the tight junction protein ZO-1, a marker for the bile canalicular membrane (Figure 2B). Faint expression was also noted at cellular borders lacking ZO-1 staining (Figure 2B, *arrowheads*). In contrast to PPL, the bile canalicular MDR (P-glycoproteins) were almost exclusively found in areas surrounded by ZO-1 (Figure 2C). Thus, PPL localizes not only near the bile canaliculi but also near other cellular boundaries of hepatocytes. The expression of PPL partially overlapped with that of the desmosomal protein γ-CTN (Figure 2D). Similar partially overlapping expression of PPL with desmosomal protein has also been reported in cultured keratinocytes [13]. Importantly, PPL expression was undetectable by immunohistochemical analysis in *Ppl*^−/−^ mice, thus demonstrating antibody specificity (Figure 2E). In line with these results, human *PPL* mRNA expression was also found to be high in a cholangiocyte-derived cell line (HuH7), considerably lower in a hepatocyte-derived cell line (HepG2), and almost undetectable in a hepatic stellate cell-derived cell line (LX-2) (data not shown).

### PPL accumulated at the boundaries of hepatocytes during dietary CA-induced cholestasis

**Figure 3:**
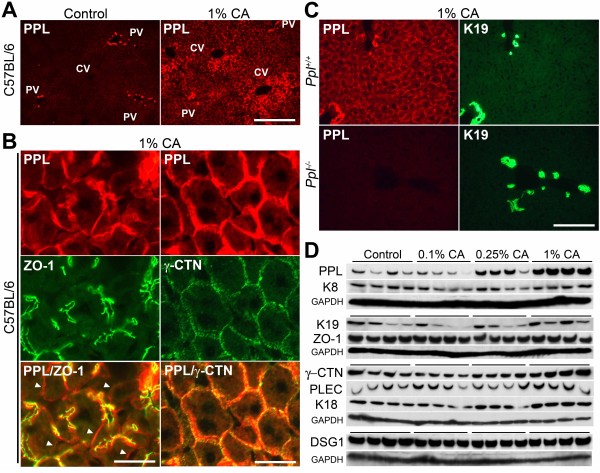
**Immunohistochemical localization of PPL in the cholestatic liver.** (**A**) Representative images for the hepatic localization of PPL in mice fed the control or 1% CA-containing diet for 7 days. PPL was detected by red fluorescence. Scale bar: 100 μm. (**B**) PPL and ZO-1 (*left column*), or PPL and γ-CTN (*right column*), were double-stained by red and green fluorescence, respectively. Merged images are shown at the bottom. Arrowheads indicate PPL expression at ZO-1-negative sites (*left bottom*). Scale bar: 25 μm. (**C**) PPL and K19 were double-stained by red and green fluorescence, respectively. Signals for PPL were absent in cholangiocytes and hepatocytes of 1% CA-fed *Ppl*^−/−^ mice. Scale bar: 100 μm. (**D**) Protein abundance of the selected hepatic structural molecules in the liver of CA-fed mice at indicated doses for 7 days. Four mice per group were examined by western blotting analysis. The immunoblots for GAPDH were used as the loading controls. PV, portal vein; CV, central vein.

The expression of PPL during dietary CA-induced cholestasis was determined by immunohistochemical examination in wild-type mice. The immunoreactive PPL expression was not increased in mice fed the lowest concentration of CA (0.1%), while it slightly increased in mice fed 0.25% CA (data not shown). In contrast, a striking increase in PPL expression was found in mice fed the highest concentration of CA (1%; Figure 3A). The increase was primarily observed at the cellular boundaries of hepatocytes. PPL expression was primarily increased in ZO-1-positive locations; however, its expression was also increased in ZO-1-negative sites (Figure 3B, *left*). Thus, in response to dietary CA, PPL appeared to form an impressive web-like structure throughout the entire liver (Figure 3A, *right*). This type of distribution was reminiscent of a similar reticular localization pattern for PPL reported in epithelial tissues, including the epidermis, cervix uteri, and esophagus, under normal conditions [13,25]. PPL expression partially overlapped with that of γ-CTN following dietary CA, similar to what was observed in the basal state (Figure 3B, right). Importantly, the immunoreactivity of PPL was completely abolished in CA-fed *Ppl*^−/−^ mice (Figure 3C). The marked accumulation of protein after CA feeding was selective to PPL and was not observed for other structural molecules, including plakin, junctional protein, and cytoskeletal components examined (Figure 3D).

**Figure 4:**
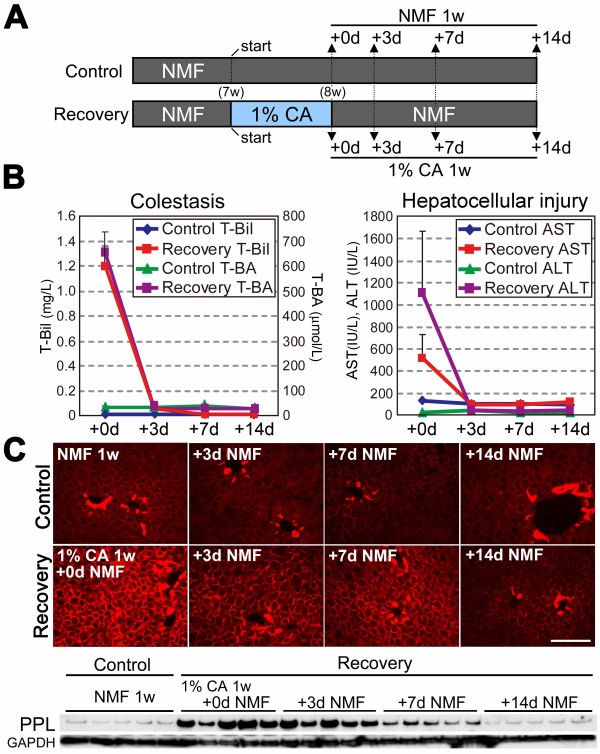
**PPL expression after recovery from dietary CA-induced cholestasis.** (**A**) Dietary administration schedule. Seven weeks-old male wild-type mice fed NMF diet were separated in two groups. One group (*Recovery*) was given the 1% CA-containing NMF diet for 1 week to induce cholestasis; the diet was then replaced by the control NMF diet to resolve the cholestatic situations. The other group (*Control*) was given the control NMF diet throughout the experiment. Mice were sacrificed in each group at the time points indicated by arrows. (**B**) The levels of the serum markers for cholestasis (T-Bil and T-BA; *left panel*) and hepatocellular injury (ALT and AST; *right panel*) at each time point in control and recovery groups. Values are shown by the symbols and colors as indicated in inlets. Averages from 5 mice are expressed with standard errors. (**C**) Hepatic PPL expression examined by immunohistochemical and western blot analyses. Representative images resulting from immunohistochemical analysis are shown for each group (*upper*). Five mice per group were examined for western blot analysis (*lower*). The immunoblot for GAPDH was used as the loading control. Scale bar: 100 μm.

When the diet was switched back to the normal diet after the induction of cholestasis by the administration of dietary CA for 7 days (Figure 4A), the levels of serum markers indicative of cholestasis, i.e., total bilirubin (T-Bil) and total bile acids (T-BA), and those of markers for hepatocellular injury, i.e., alanine aminotransferase (ALT) and aspartate aminotransferase (AST), rapidly declined to the normal range within 3 days (Figure 4B). Under these conditions, the amount of accumulated PPL protein subsequently decreased (Figure 4C). Thus, the hepatic modulation of PPL was reversible, suggesting that the covalent irreversible crosslinking of PPL typically found in the skin and mediated by TGases did not function in the livers of these mice. In contrast to the time course for the serum markers, the decrease of accumulated PPL in the liver occurred more slowly after the reinstatement of the diet, manifesting as a complete reversion to basal levels after 2 weeks (Figure 4B and C).

### Dietary CA-induced accumulation of PPL was attenuated in the liver of *Fxr*^−/−^ mice

**Figure 5:**
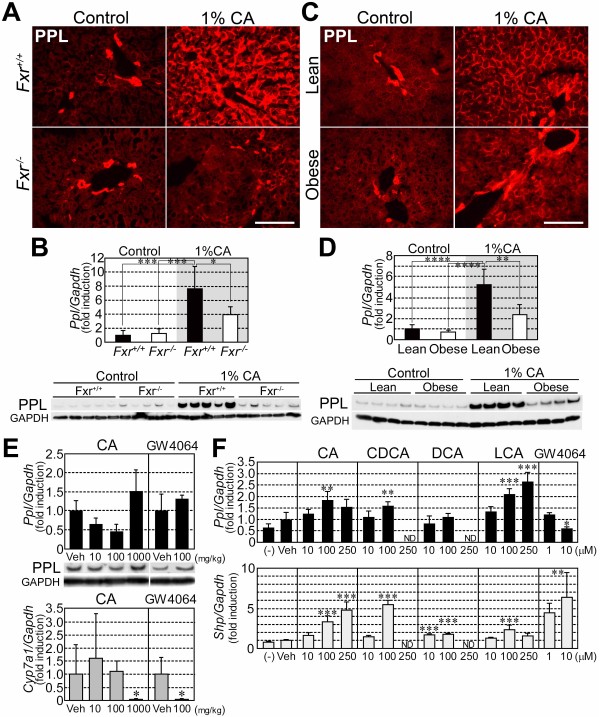
**The effect of FXR ligands on PPL expression.** (**A**) Representative images for hepatic PPL distribution in *Fxr*^+/+^ and *Fxr*^−/−^ mice fed the control or 1% CA-containing diet for 7 days. Scale bar: 100 μm. (**B**) Hepatic mRNA and protein expression of PPL in *Fxr*^+/+^ and *Fxr*^−/−^ mice under normal and CA-fed conditions. Five mice per group were examined. The immunoblot for GAPDH was used as the loading control. (**C**) Hepatic PPL distribution in NMF-fed (lean) or HFD-fed (obese) mice following CA feeding. Scale bar: 100 μm. (**D**) Hepatic mRNA and protein expression of PPL in lean and obese mice following CA feeding. Four mice per group were examined. The immunoblot for GAPDH was used as the loading control. (**E**) Hepatic *Ppl* and *Cyp7a1* mRNA expression levels in mice acutely administered CA or GW4064 via oral gavage at the indicated doses. Five mice per group were examined. The immunoblot for PPL is also shown. Equal amounts of protein from 5 mice were combined in each group for western blotting. The immunoblots for GAPDH were used as the loading controls. (**F**) *Ppl* and *Shp* mRNA expression levels in mouse primary hepatocytes administered FXR ligands at the indicated concentrations. Four samples per group were examined. (−), untreated; Veh, vehicle. mRNA expression levels are normalized to *Gapdh*. The average values in control diet-fed *Fxr*^+/+^ mice (**B**), lean mice (**D**), or vehicle-treated groups (**E** and **F**), were set to 1.0. Values represent mean ± SD. *, **, ***, ****: *P* < 0.05, 0.01, 0.001, and 0.0001, respectively, by one-way ANOVA with *post hoc* tests (Bonferroni’s test [B and D] or Dunnett’s test [F; vs. Veh]), Steel’s test (E [CA vs. Veh]), or unpaired *t* test (E [GW4064 vs. Veh]). ND, not determined.

The effect of CA feeding on PPL expression was investigated in the liver of *Fxr*^+/+^ and *Fxr*^−/−^ mice. PPL markedly accumulated at the periphery of hepatocytes in *Fxr*^+/+^ mice throughout the liver after CA feeding (Figure 5A, *upper right*). In contrast, the accumulation of PPL by the same treatment was significantly attenuated in *Fxr*^−/−^ mice (Figure 5A, *lower right*). Both western blotting and qPCR analyses detected increased PPL and *Ppl* mRNA expression, respectively, even in *Fxr*^−/−^ mice fed 1% CA, albeit the degree of increase was lower than that in *Fxr*^+/+^ mice (Figure 5B).

Because *Fxr*^−/−^ mice spontaneously develop fatty liver [8], we hypothesized that attenuated induction of hepatic PPL expression in *Fxr*^−/−^ mice might be derived from excessive fat deposition. To examine this hypothesis, we created an obese mouse model with fatty liver by feeding wild-type mice a HFD for 2 months. These mice were then administered a control or 1% CA-containing diet for 7 days to examine the response to cholestasis. Similar to the effects observed in *Fxr*^−/−^ mice, the induction of PPL expression in response to CA feeding was suppressed in obese mice (Figure 5C and D). Given the unaltered expression of FXR in the liver of HFD-fed mice (data not shown), the attenuated induction of PPL expression in obese mice seemed to be independent of FXR. Taken together, these data suggest that the impaired PPL accumulation in *Fxr*^−/−^ mice during cholestasis was not a direct consequence of the loss of FXR, but rather resulted from excess fat deposition in the liver.

### PPL expression was not directly regulated by FXR

To examine the relationship between FXR and PPL expression in more detail, we administered CA or the synthetic FXR ligand GW4064 acutely to wild-type mice. In contrast to chronic administration (Figure 1B), short-term (14 h) treatment with either CA or GW4064 did not cause an increase in hepatic PPL expression (Figure 5E, *upper*) despite clear evidence of FXR activation as shown by suppression of *Cyp7a1* mRNA expression (Figure 5E, *lower*). These results suggest that hepatic PPL induction was not directly mediated through activation of FXR. The effect of FXR ligands on *Ppl* mRNA expression was also examined *in vitro* using primary mouse hepatocytes. Although expression of the FXR target gene *small heterodimer partner* (*Shp*) was highly induced by bile acids and GW4064, particularly at higher concentrations (Figure 5F, *lower*), *Ppl* mRNA expression was only modestly increased by the same treatment in mouse primary hepatocytes (Figure 5F, *upper*). *Ppl* mRNA was even decreased by treatment with 10 μM GW4064. Thus, robust induction of PPL expression by bile acids required chronic treatment *in vivo*, and the conditions required for this type of regulation were not fully emulated by cultured primary mouse hepatocytes.

### PPL accumulation was associated with cholestatic injury

**Figure 6:**
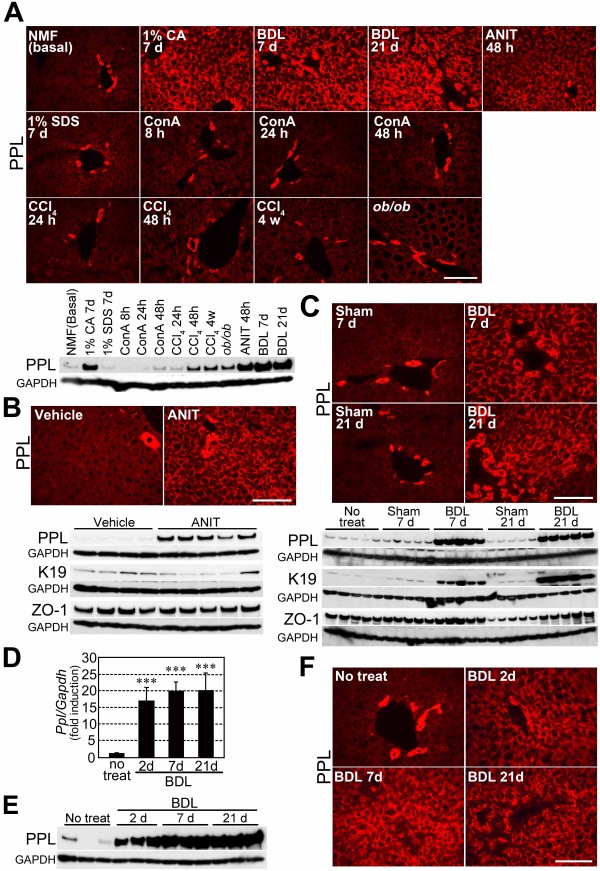
**The hepatic accumulation of PPL was highly associated with cholestasis.** (**A**) Representative images for the hepatic accumulation of PPL under different types of hepatitis mediated by the indicated triggers. Protein abundance of PPL measured by western blotting is also shown at the bottom. Equal amounts of protein from 5 mice were combined in each group for western blotting. The immunoblot for GAPDH was used as the loading control. Scale bar: 100 μm. (**B**) Hepatic PPL expression in vehicle- or ANIT-treated mice examined by immunohistochemical and western blotting analyses. Protein abundances of K19 and ZO-1 examined by western blotting are also shown. Four or 5 mice per group were examined. The immunoblots for GAPDH were used as the loading controls. Scale bar: 100 μm. (**C**) Hepatic PPL expression levels in bile duct-ligated (BDL, for indicated durations) or sham-operated mice examined by immunohistochemical and western blotting analyses. Immunoblots for K19 and ZO-1 are also shown. Five mice per group were examined. The immunoblots for GAPDH were used as the loading controls. Scale bar: 100 μm. (**D**) Time course of the induction of hepatic *Ppl* mRNA expression in wild-type mice after bile duct ligation. Five to 10 mice per group were examined. mRNA expression levels are normalized to *Gapdh* and are expressed as mean ± SD. The average value in the liver of untreated mice was set to 1.0. ***: *P* < 0.001 by one-way ANOVA with Dunnett’s test (vs. untreated). (**E**) Time course of the hepatic PPL accumulation in bile duct-ligated mice examined by western blotting analysis. Three mice per group were examined. The immunoblot for GAPDH was used as the loading control. (**F**) Representative images for the time course of the hepatic PPL accumulation in bile duct-ligated mice examined by immunohistochemical analysis. Scale bar: 100 μm.

One of the most distinctive physiological alterations following CA feeding is hepatocellular injury [31,32]. To evaluate the effects of hepatocellular injury on PPL expression, we broadly examined PPL expression in damaged liver of selected mouse models. First, we examined non-cholestatic models mimicked by a non-bile acid detergent, SDS; a plant lectin, ConA; and an organic solvent, CCl_4_. These models represent detergent-induced (SDS), hyperactive T lymphocyte-mediated (ConA), and reactive oxygen species-mediated (CCl_4_) injuries [33,34]. Although slight differences were observed depending on the doses and durations applied, all of these treatments caused similar effects on PPL expression, with incremental changes in mRNA; however, robust protein accumulation was not detected (Table 1 and Figure 6A). Second, the expression of PPL was examined in models with chronic damages from hepatic fibrosis or steatosis. CCl_4_ is a potent inducer of hepatic fibrosis when administered chronically [34]. After 4 weeks of CCl_4_ intoxication_,_ severe hepatic fibrosis accompanied by a substantial elevation in serum ALT and AST levels was induced. However, a marked increase in *Ppl* mRNA or robust accumulation of PPL protein was not observed (Table 1 and Figure 6A). Next, leptin-deficient genetically obese (*ob*/*ob*) mice were used as a model for steatosis. While a slight increase in *Ppl* mRNA expression was observed, robust accumulation of PPL protein was not detected in this model (Table 1 and Figure 6A). Lastly, expression of PPL in cholestatic mouse models was investigated using ANIT and BDL; these approaches induce 2 distinct types of cholestasis, cholangiocellular injury-derived (intrahepatic) and obstructive (extrahepatic) cholestasis, respectively [35,36]. Severe cholestasis was induced by both treatments, as confirmed by serum T-BA and T-Bil levels (Table 1). Both the increase in *Ppl* mRNA expression and the accumulation of the PPL protein at cellular boundaries were quite apparent in these models (Table 1 and Figure 6A, B, and C). Notably, the accumulation of PPL following BDL was observed as early as 2 days post-ligation, suggesting a role for PPL in early response to cholestasis (Figure 6D, E, and F). In addition, while bile duct ligation significantly increased the amount of K19, ANIT did not affect K19 expression, similar to dietary CA (Figures 6B, C, and 3F).

**Table 1:** PPL expression in experimental mouse models of hepatic injury

	**Triggers/Treatments**		**Serum markers**						**PPL**		
			**N**^**†**^	**ALT(IU**/**L)**	**AST(IU**/**L)**	**T**-**Bil(mg**/**L)**	**N**^**‡**^	**T**-**BA(μmol**/**L)**	**N**^**§**^	**mRNA(qPCR)**	Protein(IHC*)
**Dietary bile acid**	CA	0%	5	25±2	79±16	0.06±0.01	5	6.4±1.3	8	1.0±0.30	-
	(7 d)	0.1%	5	45±9	107±13	0.06±0.01	5	11±4.3	7	1.7±0.87	-
		0.25%	4	494±133	496±192	0.08±0.01	4	49±21	8	3.8±1.7	+
		1.0%	5	428±186	315±120	0.89±0.43	5	460±65	8	9.5±4.1	++++
**Dietary detergent**	SDS	0%	5	33±3	75±12	0.05±0.01	5	6.3±3.1	5	1.0±0.57	-
	(7 d)	0.1%	5	36±11	94±21	0.05±0.01	5	7.4±1.9	5	1.6±0.52	-
		0.25%	5	32±5	117±23	0.06±0.01	5	8.8±2.4	4	1.7±0.66	-
		1.0%	5	43±6	219±23	0.11±0.02	5	6.0±1.5	4	4.0±2.1	-
**Acute hepatitis**	ConA	Initial	4	33±1	107±10	0.08±0.01	4	3.1±1.3	4	1.0±0.20	-
(T cell-mediated)		Veh 8 h	6	34±8	111±25	0.08±0.01	6	3.6±0.76	6	2.8±0.73	-
		ConA 8 h	5	4927±3414	3181±2121	0.55±0.10	5	27±14	5	3.5±1.9	-
		Veh 24 h	5	38±8	120±30	0.08±0.01	5	6.2±2.6	5	1.3±0.76	-
		ConA 24 h	4	6866±813	6296±1025	0.23±0.06	4	42±9.1	4	6.7±3.4	+
		Veh 48 h	5	35±6	110±28	0.05±0.01	5	3.2±0.78	4	1.6±0.23	-
		ConA 48 h	8	376±201	423±124	0.10±0.02	8	14±6.8	8	2.9±0.61	+
**Acute hepatitis**	CCl_4_	Veh 24 h	5	42±25	169±43	0.08±0.02	5	2.4±0.53	5	1.0±0.23	-
(ROS-mediated)		CCl_4_ 24 h	5	7774±2745	5235±1775	0.21±0.03	5	69±15	5	2.0±0.94	-
		Veh 48 h	5	36±3	117±32	0.06±0.01	5	3.3±0.90	5	1.1±0.42	-
		CCl_4_ 48 h	5	11376±2964	7140±2472	0.19±0.03	5	120±20	5	7.6±1.8	+
**Hepatic fibrosis**	CCl_4_	Veh 4 w	5	49±19	173±50	0.06±0.02	5	2.7±0.30	5	1.0±0.69	-
		CCl_4_ 4 w	5	346±144	322±39	0.09±0.02	5	9.8±1.5	5	1.4±0.30	+
**Hepatic steatosis**	*ob*/*ob*	+/+	5	23±3	71±8	0.04±0.01	5	2.2±1.5	5	1.0±0.48	-
		*ob*/c*ob*	5	145±44	139±27	0.04±0.01	5	11±6.2	5	1.7±0.43	-
**Acute cholestasis**	ANIT	Veh 48 h	7	36±8	115±42	0.04±0.01	4	5.2±1.1	4	1.0±0.54	-
(Intrahepatic)		ANIT 48 h	5	1807±811	2572±1848	1.2±0.88	5	710±300	5	25±5.0	++++
**Chronic cholestasis**	BDL	No treat	5	22±4	77±6	0.07±0.03	3	4.5±2.4	5	1.0±0.33	-
(Obstructive)		Sham 7d	5	26±4	82±10	0.05±0.01	5	5.1±3.2	5	0.60±0.29	-
		BDL 7d	5	880±316	936±339	12.4±3.6	5	800±210	5	12±1.9	+++++
		Sham 21d	5	27±7	99±27	0.05±0.01	5	6.0±4.0	5	0.99±0.42	-
		BDL 21d	5	604±378	1174±757	10.3±2.5	5	1100±480	5	5.1±1.7	+++++

PPL expression in *Fxr*^−/−^and CA-fed cholestasis model mice.PPL accumulation at hepatocellular boundaries is expressed as the degree of enhancement of immunohistochemical staining from the basal status as shown in Figure 6C. At least 4 mice per group were used for immunohistochemistry (IHC) and blind tests by multiple investigators. Western blot analysis was used for confirmation. ^†^Number of mice used for the determination of serum ALT, AST, and T-Bil levels; ^‡^number of mice used for T-BA; ^§^number of mice used for qPCR. Values represent mean ± SD. ALT, alanine aminotransferase; ANIT, α-naphthylisothiocyanate; AST, aspartate aminotransferase; BDL, bile duct ligation; T-BA, total bile acids; T-Bil, total bilirubin.

Taken together, these results indicate that hepatic *Ppl* mRNA expression could be elevated by several distinct mediators of hepatocellular injury; however, robust induction at the protein level and accumulation at hepatocellular boundaries occurred only after treatments that cause cholestasis. Because many of the employed treatments (ConA [8 h and 24 h], CCl_4_ [24 h and 48 h]), elicited greater hepatic damage than 1% CA feeding (Table 1), it was evident that the severity of hepatocellular injury was not the key determinant for the degree of PPL accumulation; rather, the difference between cholestatic and non-cholestatic responses was of prime importance.

### PPL accumulated in renal tubular epithelial cells following ureteral obstruction

**Figure 7:**
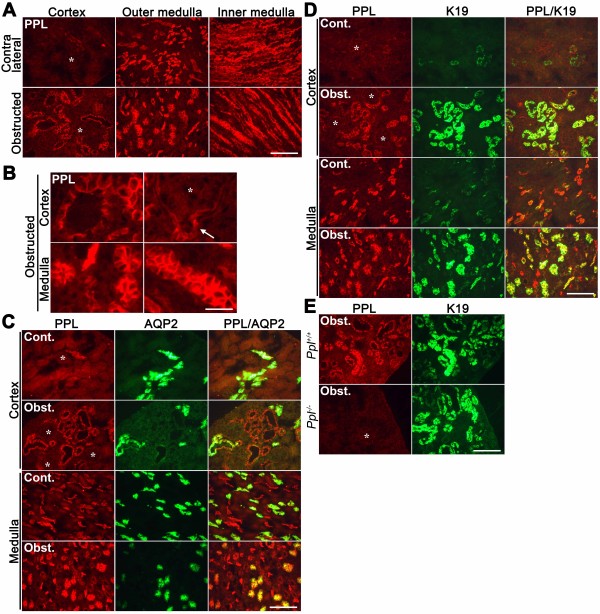
**Induction of the renal accumulation of PPL near the tubules of obstructed kidneys.** (**A**) Representative images for the localization of PPL in the cortex, outer medulla, and inner medulla of the contralateral (unobstructed) and obstructed kidneys. Results from wild-type mice subjected to unilateral ureteral obstruction for 7 days are shown. PPL was detected by red fluorescence. Scale bar: 100 μm. (**B**) Magnified images for PPL localization in the cortex and medulla of obstructed kidneys. The arrow indicates the urinary pole of the corpuscle. Scale bar: 25 μm. (**C**) Partial overlapping of the localization of PPL and aquaporin 2 (AQP2) in the cortex and outer medulla of contralateral (Cont.) and obstructed (Obst.) kidneys. PPL and AQP2 were double-stained by red and green fluorescence, respectively. Merged images are shown on the right. Scale bar: 100 μm. (**D**) Partial overlapping of the localization of PPL and K19 in the cortex and outer medulla of contralateral and obstructed kidneys. PPL and K19 were double-stained by red and green fluorescence, respectively. Scale Bar: 100 μm. (**E**) Immunohistochemical signals for PPL were absent in the obstructed renal cortex of *Ppl*^−/−^ mice. Scale bar: 100 μm. Asterisks in each panel indicate glomeruli.

To discriminate between the effects of the flooded bile in the liver and those mediated by fluid obstruction in general on PPL accumulation, we investigated renal PPL expression during ureteral obstruction. PPL expression in tubular epithelial cells in the renal cortex was markedly increased in wild-type mice after ureteral obstruction for 7 days (Figure 7A and B). Similar to its accumulation in hepatocytes, PPL was condensed at the cellular periphery (Figure 7B). Weaker induction was also noted at Bowman’s capsule and contiguous proximal tubules at the urinary pole of the corpuscle (Figure 7B, *arrow*). In contrast to the observation in the liver, robust accumulation in the renal cortex was not recognizable by day 2 of the obstruction (data not shown). The expression of PPL partially overlapped with that of the collecting duct marker AQP2 (Figure 7C) [37] and almost completely overlapped with that of K19 in the renal cortex (Figure 7D); K19 is predominantly expressed in the distal tubules and collecting ducts of normal kidneys [38]. The expression of PPL and K19 in the renal cortex occurred synchronously, i.e., very low at the basal state and markedly increased after ureteral obstruction (Figure 7D). A similar synchronous increase of PPL and K19 was also observed in cholestatic mice produced by BDL but not in other models (i.e., CA feeding and ANIT intoxication; Figures 3F, 6B, and 6C). Thus, a cooperative function of PPL and K19 may be observed in severely obstructed tissues. The specificity of the immunohistochemical signal for PPL was also confirmed in the kidney by using *Ppl*^−/−^ mice (Figure 7E).

## Discussion

The current study demonstrated that a plakin family cytolinker protein, PPL, whose molecular function *in vivo* is still largely unknown, dramatically accumulated in the cholestatic liver and obstructed kidney. The hepatic accumulation of PPL was observed as an early response to cholestasis; it was detectable as early as 2 days post-BDL and even in a milder cholestatic mouse model lacking the hallmarks of severe obstructive injury (i.e., dietary CA administration). On the other hand, the accumulation of PPL persisted more than 1 week after the removal of cholestatic agents, while recovery from hepatocellular injury was observed within a few days. This rapid-onset and long-lasting mode of PPL accumulation suggests the potential involvement of PPL in liver protection and repair. In addition, the robust accumulation of PPL found at the periphery of renal tubules following urinary obstruction suggests a similar role for this protein in the liver and kidney.

Importantly, in contrast to the irreversible incorporation of PPL into the CE layer of epidermis through covalent crosslinking by TGases, the accumulation of PPL at the cellular periphery of hepatocytes occurred in a reversible manner, suggesting a different mode of usage for PPL in cutaneous and non-cutaneous tissues. Intriguingly, other studies recently revealed that the distribution of PPL at cellular peripheries, which was observed in normal epithelial tissues, is markedly reduced during tumorigenic progression in the esophagus [25]. Thus, the peri-plasma membrane distribution of PPL may be important for protecting cells not only from the damages caused by fluid obstruction, but also from tumorigenesis. It is tempting to assume that PPL functions through a bidirectional shift between the basal and accumulated state at cellular boundaries, depending on the timing and context in different epithelial tissues. Apart from indicating the physiological roles for PPL, our results suggest that the intracellular accumulation of PPL might serve as a novel biomarker for fluid obstruction, because its abundance and intracellular localization clearly reflect the presence of the pathological state.

The precise molecular mechanism underlying the initiation of PPL accumulation during cholestasis is not fully understood. One possibility is that the accumulation of PPL is triggered by biliary constituents building up in the liver. However, because short-term administration of CA failed to elicit robust PPL expression both *in vivo* and *in vitro*, CA may not be an immediate trigger. Other factors that accumulate in the cholestatic liver and/or the physiological alterations accompanying cholestasis may also be important. Notably, the mRNA and protein levels of PPL were not completely paralleled (Table 1), suggesting the importance of post-transcriptional regulations of PPL expression in response to cholestasis. The marked accumulation of PPL in the renal cortex during urinary stasis suggests that structural and/or physiological alterations common to the liver and kidney, such as increased hydrostatic pressure or mechanical stretch, might be of importance for the accumulation of PPL. Indeed, recent studies revealed that the expression of PPL can be highly induced by biomechanical stress in saphenous vein coronary artery bypass grafts and in cultured cells grown under a cyclic stretch [39]. However, possibilities still remain that the increase in intra-tissue or systemic humoral factor(s) such as the immunological factors, would be the common trigger for PPL accumulation in the liver and kidney.

Despite the fact that our study was based on microarray analyses of *Fxr*^−/−^ mice, the regulation of PPL expression via FXR appeared to be indirect. Indeed, hepatic *Ppl* mRNA expression was lower in *Fxr*^−/−^ mice at the basal state, and the accumulation of PPL following bile acid feeding was clearly attenuated in the liver of *Fxr*^−/−^ mice. However, several lines of evidence support the indirect regulation of PPL expression through FXR: (i) acute administration of synthetic FXR ligand did not induce *Ppl* mRNA expression *in vivo* or *in vitro*; (ii) the attenuated accumulation of PPL during cholestasis in the liver of *Fxr*^−/−^ mice appeared to be secondary to fat deposition; and (iii) the proximal promoter region of mouse *Ppl* gene, that was deduced from the human *PPL* gene [40], does not contain typical FXRE (data not shown).

Based on recent results of chromatin immunoprecipitation coupled with genome-wide sequencing (ChIP-seq), there is a potent FXR-binding site 4.3 kb upstream from the transcription start site of the mouse *Ppl* gene [41,42]. We examined whether this site was a functional FXR response element (FXRE) by using conventional ChIP assays in the liver of mice treated with FXR ligands (CA and GW4064). Although basal binding of FXR to this site was confirmed, increased binding of FXR to the same element was not observed. In clear contrast, binding of FXR to the canonical FXRE in the *Shp* promoter was dramatically increased by FXR ligands (data not shown). These results suggest that the potent FXR-binding site found in the *Ppl* promoter is different from the canonical FXRE. This unique element in the *Ppl* promoter might be responsible for the enigmatic mode of regulation of the hepatic PPL expression at least in a part.

## Conclusions

In conclusion, PPL emerged as an intriguing unique molecule that dramatically accumulated at the cellular boundaries of hepatocytes and renal tubular epithelial cells in response to biliary and urinary obstruction. Because a similar modal shift in PPL expression has also been observed during tumorigenesis in epithelial tissues, further examination of the role of PPL may lead to the discovery of a novel mechanism for cellular protection by cytolinkers that is applicable to many tissues and in many contexts, including cholestasis and tumorigenesis.

## Abbreviations

ALT: Alanine aminotransferase; ANIT: α-Naphthylisothiocyanate; AQP2: Aquaporin 2; AST: Aspartate aminotransferase; BDL: Bile duct ligation; CA: Cholic acid; CDCA: Chenodeoxycholic acid; ChIP-seq: Chromatin immunoprecipitation coupled with genome-wide sequencing; CE: Cornified envelope; K: Keratin; ConA: Concanavalin A; γ-CTN: γ-Catenin; DCA: Deoxycholic acid; EVPL: Envoplakin; FXR: Farnesoid X receptor; FXRE: FXR Response element; GPBAR1: G-Protein-coupled bile acid receptor 1; IVL: Involucrin; LCA: Lithocholic acid; MDR: Multidrug-resistance protein; PPL: Periplakin; qPCR: Quantitative RT-PCR; Shp: Small heterodimer partner; T-BA: Total bile acids; T-Bil: Total bilirubin; TBST: Tris-buffered saline + 0.05% Tween-20; TGase: Transglutaminase; UUO: Unilateral ureteral obstruction; ZO-1: Zonula occludens-1.

## Competing interests

The authors declare that they have no competing interests.

## Authors’ contributions

SI conceived the study and participated in coordination of the studies. SI and JS participated in most of the animal studies, gene expression analyses, immunoassays, and drafting of the manuscript. TM participated in the gene expression analyses. YS participated in ChIP assays and the assays using *Fxr*^−/−^ mice. SA participated in the assays using *Fxr*^−/−^ mice. CA and WS participated in the CCl_4_ intoxication studies. JP and FG participated in coordination of the studies and helped to draft the manuscript. All authors read and approved the final manuscript.

## Pre-publication history

The pre-publication history for this paper can be accessed here:

http://www.biomedcentral.com/1471-230X/13/116/prepub
